# Identifying the underlying challenges that face doctoral education in chemistry

**DOI:** 10.1371/journal.pone.0322446

**Published:** 2025-05-21

**Authors:** Melissa A. Collini, Benedicta Donkor, Jordan Harshman

**Affiliations:** Department of Chemistry and Biochemistry, Auburn University, Auburn, Alabama, United States of America; Woldia University, ETHIOPIA

## Abstract

Doctoral education in chemistry (DEC) in the United States is charged with producing scientists who are capable of addressing the world’s Grand Challenges, enhancing quality of life and innovation both domestically and globally through advanced science. However, many believe these doctoral programs are failing to adequately and equitably prepare students for those responsibilities. While numerous challenges have been identified, many are based in perspective and opinion rather than inferred from theory-driven education research. This is problematic as it does not give evidence-based insight into the challenges facing DEC. This qualitative research study aims to address this issue by answering the research question: What are the issues and challenges within doctoral education in chemistry from the faculty perspective? This will be accomplished by interviewing faculty members of chemistry PhD programs and analyzing these interviews to characterize the challenges undermining DEC in the United States. Our findings indicate that there are four main themes characterizing these challenges: 1) universities and faculty struggle to find **balance** between multiple responsibilities; 2) there are no standard or robust **assessments** to assess student outcomes; 3) the **implementation** of many programmatic elements is ineffectual; and 4) inadequacies and inconsistencies with **mentorship** are deeply problematic. Research implications for these findings are significant as they give insight into the underlying, systemic challenges that face DEC, rather than simply identifying symptomatic, surface-level issues. This lays the foundation for future research addressing challenges facing DEC. Our results are presented to equip those looking to reform doctoral education with essential insights needed to understand and begin addressing the aforementioned areas of concern.

## Introduction

Doctoral education in chemistry (DEC) in the United States is charged with producing scientists who are capable addressing the world’s grand challenges, enhancing quality of life and innovation both domestically and globally through advanced science [1]. Yet, there is growing concern that DEC is failing to adequately and equitably prepare students for these responsibilities in the workforce [2–9]. Evidence indicates students who complete their training in DEC feel underprepared for their responsibilities both in academia and industry, while another 38% of students do not ever complete their training [3,10–12]. Further, systemic injustice has become a growing concern in the scientific community, a concern which has been echoed in chemistry, with recent estimates showing more than 90% of domestic students in DEC in the United States are white and more than 70% are male [13–22]. As chemistry is foundational to industries such as technology, medicine, and food, this failure to adequately and equitably prepare scientists for their potential roles risks slowing societal progress, limiting creativity and innovation, and thereby preventing us from generating better solutions to the world’s largest problems [23].

Many have opined solutions to these and other problems in DEC, but, much like finding a cure for a disease, research identifying the underlying causes of these issues is needed prior to offering solutions. Currently, primary challenges and proposed solutions are typically based in anecdotal experiences and opinion rather than inferred from theory-driven education research [2]. This is problematic, as there is no clear way of assessing if these proposed issues are actually the underlying cause of failure to adequately and equitably prepare students. Without clear problems, there are not clear solutions. Only with robust, empirically identified issues will the community be able to propose the warranted systemic changes to DEC programs.

Thus, the objective of this research is to address this problem by providing an evidence-based diagnosis of what issues lead to the systemic lack of equitable and effective training.

### Theoretical frameworks

This research was in alignment with the cognitive apprenticeship theory [24–28] of graduate education and teacher-centered systemic reform (TCSR) change theory [29–31]. Cognitive apprenticeship theory of graduate education is the understanding that students develop expertise by learning from an expert [24–28]. In DEC, doctoral advisors are considered experts and the graduate students are considered apprentices [32]. The theory states students need to learn information and *processes* by which an expert does science [24–26]. Cognitive apprenticeship mandates the expert makes visible and attainable the “invisible” processes like critical thinking and problem-solving [25]. If we are to understand the difficulties facing the development of those students, the person primarily responsible for their training is the most central agent and focus of graduate training according to the cognitive apprenticeship theory.

As this project seeks to identify factors implicated in systematic reform of DEC, advisors’ primacy in DEC makes their perceptions paramount to understand [29–31]. TCSR is a model for education reform which holds the instructor’s personal factors (identities, background, experiences), beliefs, and the context are all interrelated with one another and with the resulting practice (and therefore reform) [29–31]. This change theory provided a framework for the design of this study: to understand the primary challenges of DEC that will most inform reform efforts, we investigate faculty advisors’ personal factors, beliefs, and contexts related to doctoral education.

#### Purpose of the study.

This research was driven by the following research question: **What are the issues and challenges within doctoral education in chemistry from the faculty perspective?** Answers to this research question provide an evidence-centered look at the primary challenges of DEC in contrast to most extent literature which are derived from commentary and expert panels [2]. This study is one part of a larger-scale project which looks at the current state of DEC wholistically [33]. However, the present manuscript will attempt to answer only the research question listed above.

## Methods

### Positionality

The authors of this paper acknowledge that as research scientists our backgrounds, beliefs, ideas, and identities inherently impact the research projects we design, data collection, data analysis, and ultimately the conclusions we draw in both qualitative and quantitative research [34]. This is especially true when researching subject matter which is so deeply relevant to the authors, all of whom currently exist within the DEC system. The authors understand that one way to mitigate the impact of these identities is to recognize them and to actively work against allowing their influence on the research process. To that end, several of our methods were designed to minimize the impact of our identities, experiences, and biases on our research study. Those methods included: all authors attended a workshop on positionality and developed positionality statements; informal member checking was used during interviews if a statement was ambiguous; during coding, MAC and BD spoke explicitly and created memos noting biases prior to coming to consensus [34–36]. Although it is impossible to entirely remove our perspectives from this project, we believe that these methods lend trustworthiness and credibility to the data and analysis, and that the conclusions we have presented in this paper are reflective of the data and not our personal perspectives and beliefs.

### Limitations and participant selection

The most significant limitations present in this study concern participant selection. Though true for any human subject research study, participants may or may not choose to respond, resulting in a naturally self-selecting participant sample. In the case of interviewing faculty members within the tenure system, power dynamics and systemic inequality likely lead to some faculty opting out due to fear of repercussions. This concern was supported by multiple participants within the Associate Professor rank, who requested that the research team confirm anonymity, for fear of being “outed”. Additionally, the diversity of the sample was limited by the systemic inequity causing underrepresentation of many groups at the faculty level [15,23,37]. To combat this, the research team used a semi-purposive sampling method aiming to get a variety of perspectives with respect to gender, race and ethnicity, rank, and division within chemistry [38,39].

**Fig 1:**
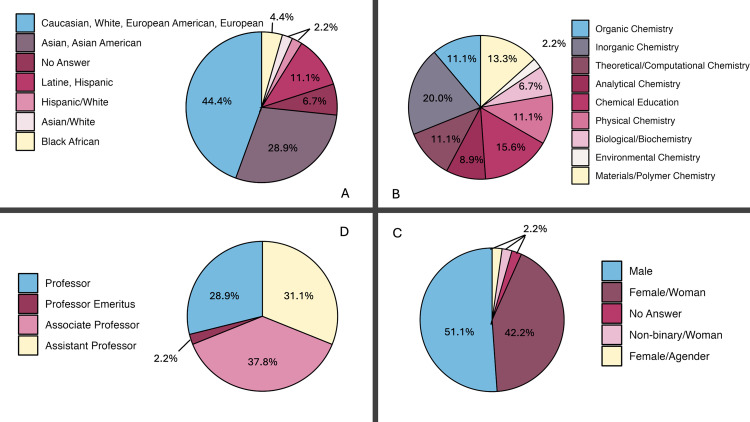
An overview of the of demographics of participants (*N* = 45). *Going clockwise from top left:* A: Demographic breakdown by self-described race/ethnicity (commas indicated groups were combined, slashes indicate mixed identities); B: Demographic breakdown by division within chemistry; C: Demographic breakdown by self-described gender; D: Demographic breakdown by academic rank.

After approval by the internal review board at Auburn University, Participants were recruited from institutions with a variety of rankings, in a variety of locations in the United States. Recruitment was carried out in several iterations from 24/10/2022 to 19/01/2024. Participants were emailed with an invitation to fill out a general survey indicating their interest and demographic information. Participants gave written consent prior to the start of their interview. The first iteration of recruitment (conducted from 24/10/2022 to 16/12/2022) yielded seven participants. Following this round, interviewing was stopped to allow time to develop a codebook (described below) and identify any changes that needed to be made in the interview protocol. Only minimal adjustments were made, primarily re-ordering questions for clarity. A second round of recruitment and interviews (*N* = 11) was conducted from 24/01/2023 to 07/02/2023, after which the demographics of the existing sample was assessed with regards to gender, race and ethnicity, rank, and division. For the third round of recruitment and interviews (conducted from 21/09/2023 to 19/02/2024, *N* = 27), participants were purposefully selected based on their responses to the demographics survey, to ensure a varied final participant sample (*N* = 45). At this point in the analysis, data saturation was determined to be reached (details in Data Analysis section) and therefore no more data was collected, resulting in the final sample size of 45 participants across 33 different universities. Information of the final sample is represented in Fig 1.

### Interview protocol

The interview protocol was initially developed by authors JH and BD and designed to assess personal factors, beliefs, and contextual factors according to TCSR framework. The protocol was then reviewed by MAC to ensure that it covered the constructs intended before beginning the first round of interviews. For this study, the primary focus was the response to the question “Are there any pitfalls or primary challenges in your doctoral program?” However, if participants noted additional challenges throughout their interviews, those were also analyzed within the scope of this manuscript. Finalized protocol can be viewed in S1.

### Data analysis

**Fig 2:**
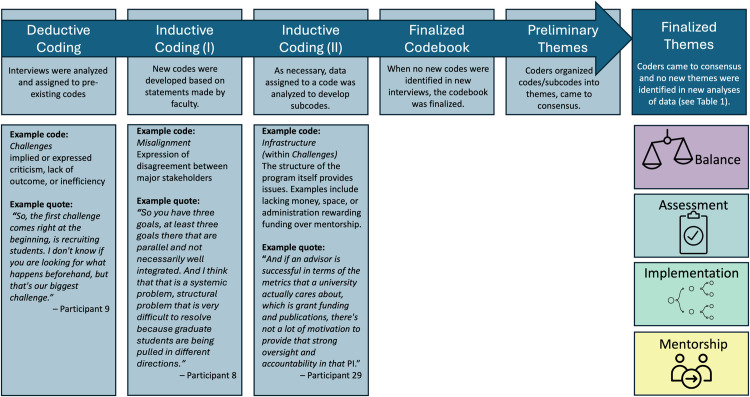
An overview of the analytical approach. This is an overview of the process which was used to identify the four final themes presented in this work, with example codes and quotes for each coding stage.

Data was analyzed by an iterative, open-coding process as there is not an existing framework regarding challenges in DEC. All coding and memoing was carried out using the qualitative coding software NVivo Version 1.7.1. Codebook was developed initially using the first seven interviews, in the process outlined in Fig 2. For each of the three coding phases, the coders (authors BD and MAC) initially coded independently, and then met to come to consensus and refine the codebook. After the initial codebook was established using a subset of the data (*N* = 7), another subset of data (*N* = 12) was coded in the same manner and the codebook was continually refined until no new codes were identified. At this stage, preliminary themes were developed. Finally, the remaining data (*N* = 27) interviews were coded independently by the coders and no new codes or themes were identified, ensuring data saturation had been reached. Inter-rater reliability for these 27 interviews was calculated Cohen’s kappa (0.74) and percent agreement (98.7%) were found appropriate to support reliable interpretation of the data. See S1 Table and S2 Table for more information regarding the codebook and codebook development.

## Results and discussion

### Overview of themes

**Fig 3:**
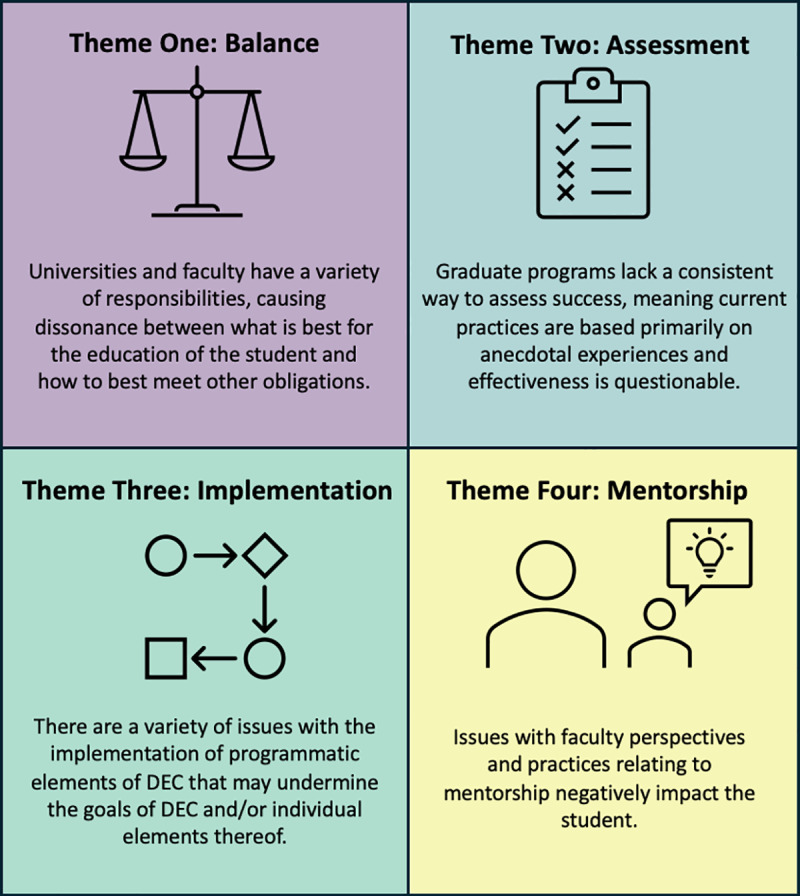
An overview of the identified themes. This is a brief overview of four themes representing the primary challenges in doctoral education. The themes have been color-coded for clarity throughout the paper. A more detailed outline of the underlying themes can be seen in Table 1.

The research question is best answered by detailing four themes that represent the primary challenges in DEC according to faculty (Fig 3). They are: (1) universities and faculty struggle to find **balance** between multiple responsibilities; (2) no standard or robust **assessment** exist to determine if DEC programs are reaching their goals; (3) poor **implementation** of existing programmatic elements leads to unrealized outcomes; and (4) inadequacies and inconsistencies with **mentorship** are deeply problematic. Due to the complex nature of DEC, there were cases in which some codes were connected to multiple themes (see S3 Table for a table which shows how each code correspond to the themes).

**Table 1:** Overview of the identified themes.

Theme	Description	Underlying ideas
**Theme One: Balance**	**Theme One: Balance **Universities and faculty have a variety of responsibilities, causing dissonance between what is best for the education of the student and how to best meet other obligations.	University responsibilities that conflict with educational goals – ranking, research mission, educating undergraduates, financial interests, etc. Faculty responsibilities that conflict with educational goals - pursuing tenure and funding, teaching courses, department service, etc.
**Theme Two: Assessment**	**Theme Two: Assessment **Graduate programs lack a consistent way to assess success, meaning current practices are based primarily on anecdotal experiences and effectiveness is questionable.	No consistent or robust way to define or assess success at the overall or individual programmatic element level Current practices modifications are made primarily on faculty anecdotal experiences and feelings DEC may not be meeting its goals, so students may not effectively be prepared for their careers
**Theme Three: Implementation**	**Theme Three: Implementation **There are a variety of issues with the implementation of programmatic elements of DEC that may undermine the goals of DEC and/or individual elements thereof.	Some programmatic elements are not being executed effectively or at all Variability in how elements are implemented causes inconsistencies in students’ training Faculty are unaware of/disagree with how their colleagues implement DEC elements Implementation of some elements place excessive burden on students
**Theme Four: Mentorship**	**Theme Four: Mentorship **Issues with faculty perspectives and practices relating to mentorship negatively impact the student.	Faculty are not explicitly (or not adequately) trained in mentorship Students are not getting enough support (cognitive and affective) from mentor Mentor-mentee relationships can degrade Mindsets about students of some faculty are deeply problematic

An overview of the themes identified and presented in this section. The left column provides the theme keyword, the middle column describes the theme, and the right column details undlying ideas associated with each theme.

**Theme One (Balance): Universities and faculty have a variety of responsibilities, causing dissonance between what is best for the education of the student and how to best meet other obligations.**

Despite the importance of graduate education in academia, there is a consistent tension between what is optimal for the students’ training and other responsibilities for universities and faculty. At the university level, other priorities include university statistics-centered incentives, financial resources, educating undergraduates, and even focusing on robust research generation can be to the detriment of educating graduate students. Participant 8 recognizes aspects of this tension:

***Participant 8:*** *“I think that there is structural issues that are complicated to resolve that. So, for example, the fact that many of our grad students have to be teaching assistants so there is this marrying of activities that are not necessarily aligned, the teaching component and the research component. And three, the research faculty wants to do their research and they want the graduate students to be their workforce. The university wants the grad students to teach their labs because they are their workforce for teaching their labs. And then the grad students would like to be prepared. So, you have three goals, at least three goals there that are parallel and not necessarily well integrated… And I would say it has been a very productive model to prepare graduate students. I’m not saying that it’s not, because clearly the United States has been very successful at creating a very strong research program with all these pitfalls.”*

In addition to the need to train undergraduates, faculty cited other university-level issues such as the devaluing of master’s degrees, the need to maintain the university’s rank, lack of space or funding to create space, and lack of funding to appropriately compensate students as issues which undermine training students.

Like the university’s many responsibilities, faculty also have conflicting demands and responsibilities that may detract from students’ training. Mentoring doctoral students (in the lab, on committees, and/or as TAs) is not the only responsibility of chemistry faculty. Participants described balancing the need to pursue tenure, pursue funding, teach courses, do departmental service, etc. They described how the goals of these pursuits may not always align with what is optimal for the student experience. Participant 14 explores this tension:

***Participant 14:*** *“So, the goal of the student and the goal of the faculty might not be perfectly aligned in that the goal of the student should be to be productive and learn the skills they need to take the next step and to learn what they need to learn and that combination of things, whereas the goal of the faculty is both having the imperative to help the students succeed but also to help their own research succeeds since they’re in the PI’s lab. So, you have a Venn diagram of the two goals, and you hope that most students and most PIs understand where those overlaps are and they try and maximize them. But when you look across the country, not everybody does that.”*

These statements demonstrate an inherent misalignment between what is optimal for the advisor and what is optimal for the student. Considering these varying responsibilities, many participants indicate that they “hope” for an ideal situation that “in theory” allows students to be successful regardless of the many responsibilities conflicting with their training which is consistent with a previous study where there is discrepancy between stated goals and enacted practices [40]. Given the high attrition rates and history of inequity in DEC [10,11,17], we are left to wonder what potential has been lost when students’ advisors fail to strike this balance.

**Theme Two (Assessment): Graduate programs lack a consistent way to assess success, meaning current practices are based primarily on anecdotal experiences and effectiveness is questionable.**

Faculty perspectives gathered in this project make it clear that there is not an agreed-upon method to assess success in DEC, both for the overall program level and individual elements, which causes problems in doctoral education, especially with students’ employability after graduation. Further complicating the issue of assessment is the lack of a clear goal as a benchmark for success in DEC. As discussed extensively in work stemming from the same interviews discussed here [33], many faculty struggled to define the goal which was ostensibly guiding their students’ training. For example, some faculty expressed hesitation when asked to state what the goal of their program was, as with participant 9:

***Interviewer:*** *“So, what are the primary goals of your doctoral program?”*

***Participant 9:*** *“Yeah, so I was going to try to find our official learning goals, but that’s not happening here. Sorry, I’m looking at some of our text right now. Oh, wait, let me just try to find something really quick here. I was trying to see if I could find something official for you, but basically we’re looking to help students develop their expertise, both kind of a breadth of expertise and a depth of expertise. And so that’s, I would say, one of our goals. The second major goal, I would say, is to help students become independent learners.”*

Other faculty stated that the goal of graduate education was to “become an expert” or become an “independent scientist”, which is in line with the ACS goal of doctoral education in chemistry, but when probed deeper, the faculty was not always able to expand the definition. For example, when asked to articulate what becoming an expert in the field meant, Participant 6 stated that there was no right definition for that.

***Interviewer*** *:” And [when asked about the goal of doctoral education] you said to become experts in their specific field, so do you have thoughts on what makes an expert?”*

***Participant 6*** *: “Oh my gosh, so hard questions. How do I qualify as being expert? I don’t know if there is again a right definition for that, and so I’m really expressing my point of view.”*

Without a clear goal in doctoral education, it is impossible to meaningfully measure success towards that benchmark, a theme repeated by several participants. Participant 12 illuminates this, recognizing their program does not have an assessment means, and what they do have is anecdotal:

***Interviewer*** *: “Overall, are the elements that we mentioned that is coursework, research seminar, and all the other elements, are they serving their intended purposes?”*

***Participant 12*** *: “Yeah, I think so. If you’re asking me what we could do different, or maybe that’s a different part of this conversation, I could probably wax poetically on that. But yeah, I believe that these things are helping with their intended purposes. If the next thing you’re going to ask me is how are we assessing that? I’m like, “Oh, I don’t know what they’re doing.” But I think you could assess it by looking at job placement. But all I would have there is anecdotal data. I don’t have any kind of rigorous data to support that those things are working.”*

With evidence of efficacy lacking, many changes made to the program are based on anecdotal experiences, rather than driven by measured data. Participant 14 describes how programmatic changes usually reflect opinions of the faculty and students, rather than looking into any literature or data:

***Interviewer:*** *“Do the changes that are made usually reflect evidence or some other programs, or just a general sense that the faculty has that one way would be better than the other?”*

***Participant 14*** *: “Evidence, yes. Comparison to other programs, yes. Opinions of the faculty, yes. Opinions of the graduate students, yes. I can say that, for example, when we made this qual change, we didn’t do an extensive literature search as to the effectiveness of comprehensive qualifying exams at the end of the first year and graduate outcomes. Then again, I doubt that there’s a whole lot of literature on that, on the way that we do it here to do comparison, right? The evidence, for example, we used was that, for example, we determined that the qualifying exams were largely superfluous from a judging student quality perspective and were a very large amount of stress on the students, and basically it ended up being a lot of stress and time for the students, and we didn’t think that they were learning anything.”*

Though faculty are making changes towards improved effectiveness of their programs, many are also lacking a clear goal and/or assessment by which to measure success. This leads faculty to implement changes based primarily on opinion or feelings, rather than evidence. Thus, it is difficult to know to what end or extent these changes are being made. This causes many to be concerned that DEC is not adequately equipping students for their post-doctoral careers [2,12,41].

**Theme Three (Implementation): There are a variety of issues with the implementation of programmatic elements of DEC that may undermine the goals of DEC and/or individual elements thereof.**

Doctoral education programs are made up of a number of individual elements (such as research, teaching assistantships, seminars, coursework, etc.) [2,42]. As noted in Theme Two, there is currently not a clear way to demonstrate these elements are serving their intended purposes. However, there *is* evidence that faculty believe some of these elements are *not* functioning optimally. These include the perception of elements that place excessive/needless burden on students, elements not being executed properly or at all, variability in student experiences, and faculty not being aware of or disagreeing with their colleagues’ practices.

Consistently, participants mentioned elements burdening students or causing stress for relatively little benefit. Participant 24 describes the stress placed on students by the candidacy process, and express lack of confidence regarding whether the goals are being met:

***Participant 24:*** *“So, if you were to remove any of these things, right, if you remove the original research proposal, you remove thesis background exam or other similar requirements, you could still have a really rich and meaningful PhD experience, and you probably wouldn’t have an extremely stressful month or so while you’re preparing for these hurdles. So, I’m not sure they serve the goals from a student’s perspective that we might hope. I’m not confident of that.*

Many other elements were discussed as having issues including coursework, seminar/colloquium and annual evaluations. Often, faculty described advisory committees failing to meet altogether for students’ annual evaluations:

***Participant 13*** *: [When asked about annual evaluations] They’re supposed to. Yeah. So, they’re supposed to have an annual advisory meeting. The current issue is that the compliance of that, both on the side of the students and it’s more so on the advisors, where advisors aren’t reminding their students or they don’t put value in it. And then of course, the students aren’t going to put value in it.*

Potentially underlying these issues with elements may be that faculty often indicated they were unaware of or disagree with other faculty’s practices. For example, participant 5, when asked what students gain from their courses, stated that they don’t pay attention to what their colleagues do.

***Interviewer*** *: “And that’s the goal, but what do you think students actually gain in their time in the courses?”*

***Participant 5*** *: “Well, that’s a good question. I don’t know. Yeah, that’s a tough one. What do they gain? In reality, I admittedly don’t pay attention to what my other colleagues [within the college] do.”*

Although Academic freedom among faculty members is inherent in the structure of DEC, faculty being unaware of other faculty’s practices in the classroom, as TA supervisors, or within their research groups results in a wide variability in students’ experiences. While this can be beneficial, it can also result in problematic practices. Participant 13 focuses on this phenomenon in comprehensive exams, and the struggle of determining if this inherent variability is fair:

***Participant 13:*** *“I think that the written and oral comprehensive exams, we are currently trying to figure out a better way of doing those in our own campus. And I think they have a really important role in the junction between when the students taking courses and when they then go on to pure research. And I think that’s an area that a lot of graduate programs struggle with. And we are also one of these programs that struggle with exactly what are we trying to get out of this, how do we evaluate them, how do you deal with variability? So, you have typically four faculty in a room with a student for the oral exam portion. Each of those four faculty are going to interact differently than any other group of four. And is that fair? Is that unfair? There’s so much variability. I don’t have a solution to that. That’s something that certainly we are thinking about as a department.”*

Participant 3 also questioned the fairness of the system due to lack of uniformity, noting the difference in research advisors’ students experience:

**Participant 3: “** *Its not necessarily uniform across research advisors. Some research advisors are less accommodating, I would say, than others, and so that I don’t think that things reach to the level of being actively unfair, but I would not say they’re exactly uniform either.”*

Participant 16 also commented on the variable nature of advising between groups, but focuses on some of the positive aspects of variability in the student experience:

***Interviewer*** *: “Do you think all advisors give morale supports to their students?”*

***Participant 16*** *: “So all is a strong word. All advisors are really different and choosing it, and all students are really different. So different students need that to a different degree, different students want that to a different degree. I’d say some students and some advisors are interested in a relationship that’s more like family and less professional distance, and other advisors or students want a lot of professional distance. So, I don’t think there’s any one good answer to this question. I even go further the other way, I think it’s a benefit to a whole system that there’s not one good answer. There should be a variety of kinds of advisors and a variety of kind of students, and they should do their best to find a match.”*

Despite participant 16’s emphasis on the benefits of academic freedom, the issues posed by other faculty demonstrate the difficulty in balancing variability with consistently adequate training for students. Further, the burden placed on students with excessive requirements and the expectation that they have meaningful takeaways from elements that are not being properly implemented may also contribute to inadequate preparation for graduate students in chemistry.

**Theme Four (Mentorship): Issues with faculty perspectives and practices relating to mentorship negatively impact the student.**

Every participant in this study stated that which is well supported by existing research [27–32,43,44]: research is the cornerstone of a PhD in chemistry and either explicitly or implicitly acknowledged that research advisors were inextricably linked with the research process. Beyond simply being linked, Nardo (2024) provides evidence that faculty in chemistry hold decision-making power over their student’s success beyond what is outlined in handbooks or policy [45]. However, many participants cited issues with mentorship in DEC, including the limiting training faculty receive, faculty failing to tailor their mentoring to students’ career goals, fallout from degradation in the advisor-student relationship, and faculty holding unhelpful views about the students.

Often faculty acknowledge that they have received no training for mentorship, and some state that may be a weakness for them:

***Participant 12*** *: “So one thing I guess I’d say that nobody does very well, and I’m just integrating back over all the places I’ve been, is that people aren’t trained how to be mentors really. So those of us who had really good PhD advisors and other things, we probably, without being told how to be a good mentor formally, maybe we learned how to be one just because we had a good one, and then you can also imagine the opposite happens. Right. So, I think that faculty, usually wherever you go, don’t necessarily have training on how to be good faculty once you’ve joined the faculty. And that covers lots of things. You don’t usually get training on grants accounting, you don’t usually get training also on mentoring strategies. And that’s something that maybe the whole enterprise of graduate education can think about, and you won’t get the same expectations or approaches as you go too far a field.”*

This lack of training may be the reason for the consistent idea expressed by participants that they have seen students receive inadequate support from their mentor. Similarly, faculty cite their colleagues failing to prioritize the career goals of their students. As can be seen with participant 19, who emphasizes aligning their mentorship types to their students’ goals, but note that not all their colleagues do the same:

***Participant 19:*** *“I tell my students, hey, like right now, your job is to develop yourself to do top-notch science, and we are doing that, and then I’m happy to consult you, but you choose your own path. If you don’t want to be a professor, that’s totally okay, but that’s not the norm. There’s many other professors that will groom their students to only consider academia. I see that as a problem, but I don’t know what to do about programmatically. That is a problem with the individual leading the group. Yeah.”*

Though career choice is one area where students and advisor may have conflict, there are other forms of conflict or relationship degradation that may occur, impacting the students’ progress [43]. Participant 12 describes relationships degrading between mentors and students, and the need for committees to intercede at times:

***Participant 12*** *[When asked about the primary goal of advisory committees]: “If things are not going... They become your defense against the dark arts if you have an evil advisor. I think that you hope that never happens. But if there is a giant... And by the way, sometimes falling out is mutual and nobody’s necessarily personal fault. It just things don’t work, and that’s it. And you’ve got to go your separate ways. But sometimes there’s not good. And I can’t really necessarily point to things at my school, but I hear tell of things that happen, crises at other schools and friends I had in their graduate programs. Where when I hear about that, they would make me mad…. Having a committee can help modulate the behavior of advisors and help provide a secondary ear. And just to help make sure things are going okay. Oh my God, I just heard so many stories.”*

The way that participant 12 describes the need to moderate the behavior of the advisor, but not the student, speaks to the significant power dynamic at play when it comes to student/advisor relationships [43,44]. They recognize that students are so powerless, and their advisors play such a significant role, that at times students require defending. Though participant 12 describes this as the role of the committee, participant 10 takes a different perspective, and cites the dilemma of protecting a student when it may make working relationships uncomfortable:

**Participant 10:** *But I think that the pitfalls are, they often happen when there’s a mentor-mentee degradation or something happens in that mentor-mentee relationship. And it’s really hard to address those. Because as if you’re a fellow faculty member, then that’s probably one of your colleagues and they might be senior to you. Or even if they’re junior to you, either way it can be awkward***.** *And then you have to have leeway for the fact that everyone mentors slightly differently, but there should be some kind of common agreed upon mentoring rules or policies. And currently that is left to, you run your own little fiefdom and don’t mess with other people’s little fiefdoms.*

These conflicting ideas presented by participants 12 and 10 capture a key challenge in DEC – little or no training in mentorship, primary responsibility for their student’s development, and little to no oversight for doctoral advisors results in a power imbalance that may only be checked by other faculty choosing to step in [44]. This is troubling as faculty members are often unaware of what their colleagues are doing (noted in Theme Three: Implementation). Or faculty may be aware but unwilling to intercede for fear of awkwardness or retaliation harming their own careers, ultimately leaving doctoral students unprotected (as noted in Theme One: Balance). Ironically, participant 10 notes the difficulty of navigating power imbalances with more senior faculty to justify inaction on behalf of students, who are facing arguably more significant consequences and power imbalances [43–45].

Beyond conflict with students, faculty may even hold views about students that can negatively impact their ability to mentor the students appropriately. Participants 40 describes this mindset:

**Participant 40***: I hate using this word, but they become cheaper labor after because we’re not necessarily paying for, I guess, pre candidacy prices. I don’t know.* *They talk about students sometimes like livestock and it makes me really uncomfortable, but they typically are cheaper I guess, after.*

**Interviewer** *: You said ‘sometimes they talk about the students like livestock and it makes me uncomfortable’. Is that the other faculty?*

**Participant 40** *: Yes. So, the other [] faculty, definitely, especially I think organic has a very transactional relationship with their students and how they view their time and them as resources and everything like that. … But I think they typically forget that they’re people and that this is their lives, that this is a really big... I mean, it’s not even like a year of their life. It’s like five years of their life. So, we are forming their sense of self, we’re forming their sense of who they will be and who they’re becoming and everything like that. And I just feel like we should take a little bit more time to respect that and to really conceptualize that when we’re making decisions about students.*

Participant 32 also gives examples of colleagues’ mindsets that may contribute to shortcomings in mentorship, essentially deciding that the students are at fault for not progressing:

**Participant 32***: You can see that it’s getting better and better. So, in those cases, it’s working, but you also see students where they seem to be stuck.* *And obviously, we can blame the students and say, “Well, they’re not putting effort.” But I don’t think it’s just that, because I’ve seen students putting the effort***,** *putting the hours in the lab and they don’t improve. So then in that case, I think it’s us failing them.*

**Interviewer** *: So, it’s working for students that it’s working for, but there may be students that it’s not working for and we’re not paying as much attention to them?*

**Participant 32***: Yes, right? And so many people see this as* *“Well, they were not made for a PhD.” But when we admitted them, we thought that they were. So how can we change our minds so quickly? So that’s my problem.*

Participant 14 shares the mindsets described by participant 32, describing how some students should not have been admitted in the first place:

***Participant 14:*** *Graduate student recruiting is very challenging. Recruiting admissions, and we talk with some frequency about how the curriculum that we have may interrelate with those things, but ultimately, I think we would all love to have the situation that we were recruiting a hundred percent amazing graduate students, and we wouldn’t have to think so hard about these things, right? And so, ultimately one of the big questions that we have is, okay, we have to establish... Most years we have students [who] do not make it through to the second year of the PhD program. That’s the norm, and that’s in large part because every year we take students in who should not be in the PhD program, but we are unable to determine that based on their academic records, et cetera. And so, this is the thing that gives us the most consternation is can we improve our admissions process and our recruitment process such that we don’t have to, that instead of thinking so much about when do we kick students out, which is something that occupies a lot of our time unfortunately, how do we switch the conversation to being about helping those that are here succeed to the best of their ability. Unfortunately, we don’t spend as much time on that latter part as we should because we have to spend too much time making up for the fact that our admissions criteria don’t work very well. It’s the same group of people that are responsible for both those things.*

These comments provide evidence that faculty may have fixed mindsets or biases against certain types of students, believing there are some students who can succeed in DEC and others who cannot. This is a problematic perspective considering the significant power that faculty hold in DEC, the lack of oversight or accountability for faculty, and the history of inequity in chemistry [7–9,13–20,23,46]. With unchecked biases (explicit or implicit) faculty can (intentionally or unintentionally) use their power to discriminate against or undermine certain types of students [44,45,47]. Participant 14 describes this very phenomenon happening annually; after deciding retroactively that they have admitted (from their perspective) the wrong type of student, this participant describes focusing their energy on “kicking out” those students, rather than supporting the students that they have admitted. Though this participant expresses a desire to help their students succeed, the participant appears to believe that is contingent on having (from their perspective) the right type of student. They go on to lament that they don’t spend as much time helping students currently the program succeed because their energy is focused on excluding students who (from their perspective) don’t belong. Thus, this participant is choosing to focus on excluding existing “students who should not be in the program,” rather than leveraging their power to help existing students succeed.

Ultimately, challenges with faculty and mentorship in doctoral education should be a primary concern to the chemistry community. Faculty are primarily responsible for training and determining the success of their students. Thus, in line with the theoretical framework of TCSR, working to address these challenges with the structure and quality of mentorship in DEC could have significant impacts on effectiveness and equity in DEC as well as on students’ lives. Without adequate support students are facing consequences which could impact their income, education, career, or mental health [43,44,48,49]. Yet, there is not a system in place for faculty training or accountability.

## Conclusions and questions

This research study aimed to answer the research question: **What are the issues and challenges within doctoral education in chemistry from the faculty perspective?** Our findings indicate that there are four main themes characterizing these challenges: (1) universities and faculty struggle to find **balance** between multiple responsibilities; (2) there are no standard or robust **assessments** to measure student outcomes; (3) the **implementation** of many programmatic elements is ineffectual; and (4) inadequacies and inconsistencies with **mentorship** are deeply problematic. These findings are significant as they give insight into the underlying, systemic challenges that face DEC, rather than simply identifying symptomatic, surface-level issues.

Our findings provide crucial insights for those aiming to reform doctoral education, highlighting key areas of concern. Yet, given the systemic nature of these problems and the necessity of individualized training in DEC, addressing these challenges can be complex. How can we as a community maintain academic freedom while ensuring accountability in power dynamics? How can we accommodate the need for individualized training, while also ensuring a minimum standard of preparedness? How can we best support and train faculty as they support and train doctoral students, along with many other responsibilities? The path forward is unclear, but TCSR suggests that faculty ought to be at the center of meaningful change in academia. To initiate this dialogue and empower faculty to begin addressing these issues, we have developed a set of questions for each of the four themes (Table 2) for faculty to discuss.

**Table 2:** An overview of the themes and questions for reflection to accompany each theme. These questions are included in order to empower faculty, and to initiate dialogue among faculty as a first step towards meaningful change.

Theme	Description	Questions for reflection
**Theme One: Balance**	**Theme One: Balance** Universities and faculty have a variety of responsibilities, causing dissonance between what is best for the education of the student and how to best meet other obligations.	Where is there dissonance within our program between the needs of the faculty and the needs of the student? Where is there dissonance between the needs of the university and the needs of the student? What systems of accountability can we implement to mitigate these issues? What support would the faculty need to achieve these changes?
**Theme Two: Assessment**	**Theme Two: Assessment** Graduate programs lack a consistent way to assess success, meaning current practices are based primarily on anecdotal experiences and effectiveness is questionable.	What is the goal of our program overall? Following a backward-design approach: What is the goal of each element (i.e., seminar, research, coursework)? How are they intended to contribute to the overall goal of the program? What assessments could be made to evaluate the efficacy of our program?
**Theme Three: Implementation**	**Theme Three: Implementation** There are a variety of issues with the implementation of programmatic elements of DEC that may undermine the goals of DEC and/or individual elements thereof.	In what ways are elements not being implemented as intended? What changes can be made to improve efficacy of elements? What support would the faculty need to achieve these changes? How can we ensure fairness and adequate training while still allowing for the benefits of academic freedom?
**Theme Four: Mentorship**	**Theme Four: Mentorship** Issues with faculty perspectives and practices relating to mentorship negatively impact the student.	What training for mentorship have we (the research faculty) currently received? How can training be implemented in a meaningful way? What mindsets about students do we as a faculty hold? What mindsets may be harmful to the development of our students? How can we preserve the academic freedom of the advisor while ensuring students are meeting a minimum standard of training? What systems of accountability can we implement to mitigate the power imbalance between faculty and students?

## Supporting information

S1 File S1 Table. Codebook development. S2 Table. Working codebook with definitions. S3 Table. Organization of codes into themes.
(DOCX)
