# Supplementary material for: Identifying the underlying challenges that face doctoral education in chemistry
Source: PLoS One. 2025 May 21;20(5):e0322446. doi: 10.1371/journal.pone.0322446 (PMC12094721; doi:10.1371/journal.pone.0322446)
Supplement: S1 File — S2 Table. Working codebook with definitions. S3 Table. Organization of codes into themes. (DOCX) [file pone.0322446.s001.docx]

**Interview Protocol**

Thank you for participating in this interview. Today, I’ll be asking you about the learning goals and outcomes of key elements of chemistry doctoral education. All questions are asked about “doctoral education in your department” or “your doctoral program.” Please interpret this to mean you can provide individual perspectives that may or may not be reflected by your colleagues.

The structure of the interview will be in three phases. First, I’ll ask you some overarching questions about your doctoral program. Then, I’ll ask a similar set of questions for several specific elements of your doctoral program. Finally, I’ll broadly ask how/if the elements interact with one another.

Even if I don’t get through all the planned questions I have, I’ll make sure we wrap up by [*time*] unless you say otherwise; is that okay with you?

Any questions before we begin?

**Faculty Perspective on the Elements of Doctoral Education (except program duration)**

I’ll mention the elements in chemistry doctoral education, let me know if your department has this element or not.

Elements present in interviewee’s Institution

| **Element** | **Present** | **Absent** |
| --- | --- | --- |
| Research** |  |  |
| Coursework** |  |  |
| Research Advisor** |  |  |
| Teaching assistants* |  |  |
| Seminars/colloquia* | (Please describe) |  |
| Candidacy exam/*  Qualifying exams/ Cumulative exams/ Original Research Proposals | (Please describe) |  |
| Dissertation and Defense* |  |  |
| Doctoral/advisory committees | (Please describe)  (Selection process if not mentioned) |  |
| Lab Rotations  If no, what is the advisor selection process? |  |  |
| Publication as a requirement | What is the requirement? |  |
| Group meetings |  |  |
| Annual Evaluations | (Please describe) |  |
| Program Duration |  |  |

Do you have any other elements I did not mention?

| **Question** | **Notes during Interview** | **Notes after Interview** |
| --- | --- | --- |
| **General questions** |  |  |
| What are the primary goals of your doctoral program |  |  |
| Should a PhD program prepare students for a career? Why do you think so? |  |  |
| Overall, are these elements serving their intended purposes?  Follow up - If yes – How do you know they are serving their intended purposes? |  |  |
| Follow up - If NO, Can you be specific with which one is not serving its purpose and how it can be made to serve its purpose? |  |  |
| Follow up - If NO, how can they be made to serve their purposes? |  |  |

| Any pitfalls in your graduate program/primary challenges? |  |  |
| --- | --- | --- |
|  |  |  |
| **Questions for all elements** |  |  |
|  |  |  |
| What are the primary goals of having students’ complete element? |  |  |
| What do you think students *actually* gain by completing element? |  |  |
| As a reminder, you stated the primary goals of doctoral education were to [recap goals].  How does completing element contribute to the overall goal of graduate education? |  |  |
| Who and/or what determines if a student has met the element requirements. |  |  |

| **Personal Factors** |  |  |
| --- | --- | --- |
| Do you think your perceptions about the purposes of the elements have changed over time (as you age in the profession) and/or as you see more students graduate (your own students or those you’ve been on their committees)? |  |  |
| How many students’ committees have you served on? |  |  |
| Have you participated in workshops mainly designed for you to think critically about graduate education? |  |  |
| **Contextual factors** |  |  |
| In general, to what extent does administrative policies and priorities (dean, department chair, departmental colleagues, graduate school) influence your perceptions about the elements? |  |  |

**Table 1 Codebook development**

| **Coding phase** | **Coding approach** | **Codes** |
| --- | --- | --- |
| Deductive coding: overall data | Interviews were analyzed and assigned to pre-existing codes (challenges). Coded independently by coders BD and MAC, who then came to consensus. | A-priori code:   - Challenges/Pitfalls |
| Inductive coding:  overall data | Interviews were analyzed to develop new codes. Codes were developed independently by coders BD and MAC, who then came to consensus. | Inductive codes:   - Misalignment - Meta awareness (aware of little/no training for mentorship) - No way to assess success. - “I don’t know” - Variability in students’ experiences - How changes are made |
| Inductive coding: within parent codes | As necessary, data assigned to a code (i.e. “Challenges”) was analyzed to develop new subcodes. Codes were developed independently by coders BD and MAC, who then came to consensus. | Inductive subcodes: Challenges   - Issues with faculty and mentorship - Issues with programmatic elements - Issues with graduate student recruitment - Issues with infrastructure - Excessive requirements burden students - Negative impact on students’ mental health - Master’s degree undervalued - Questionable preparation for career - Students’ intrinsic problems*** - Impacts of Covid-19***   Misalignment:   - Between stakeholders - Between what is best for students’ and other entities   “I don’t know”:   - What my coworkers are doing - How to support my claims |

***It is important to note that the codes “students’ intrinsic problems” and “impacts of COVID-19” were not included in any theme. This is because our paper is focused on the system of doctoral education and the systemic issues within that entity. Though these isolated incidence resulted in struggles or challenges that may cause students to depart doctoral education, we instead want to focus on what issues exist within the system itself that may exacerbate or generate issues with students.

**Table 2 Working codebook with definitions**

The below table is an overview of the codebook with working definitions

| Code name | Description |
| --- | --- |
| Meta awareness | When faculty are aware that they aren’t trained to be mentors, and graduate education has issues that need to be fixed as a whole. |
| Misalignment | When someone comments on the fact that the program’s goals or the department’s goals or the university’s goals, or the faculty in some cases goals are not in line or top priority to be in the best interest of the students. When stakeholders goals do not align. |
| No way to assess success | Mention of not having a way to assess success in the program/no assessments for outcomes. |
| Variability in student experiences | Any mention of students having variable experiences in the program, lack of consistency |
| “I don’t know” | When faculty members state that they don’t know, not sure, this is a tough question, or they’ve never thought about it before. |
| How changes are made | Whenever faculty talks about changing something, removing or adding an element and describe how that process takes place |
| Challenges/Pitfalls of the program | Any mentions of pitfalls of the program. These will be coded regardless of if they are explicitly mentioned when we ask the question - any challenges mentioned at all will be included. |
| Challenges child code-  Graduate Student Recruitment | Difficult to recruit students and know if they’re a good fit for the school. Issues with wide variety of students’ backgrounds. No way to assess or evaluate if they are good fits. |
| Challenges child code- Impacts of COVID19 | Mention of the impact of Covid 19 on the program |
| Challenges child code- Infrastructure | The way the program is set up presents issues for a variety of reasons. When administration rewards funding rather than mentorship, elements overall not set up in a way that enriches one another, not enough resources, money, space, not enough people to teach classes, etc. This includes issues with how research is funded. |
| Challenges child code- Issues with elements | When an element isn’t serving its purpose, or some other issue (not valuable, etc). Difficult to assess if they are meeting their intended purposes. Issues with advisors go here and also with faculty/mentorship |
| Challenges child code- Issues with Faculty and Mentorship | The faculty are stuck in the past, not willing to update teaching or mentorship practices, there is variety in the program, there is not training for mentorship. There are also issues with no standardized assessments between professors, inconsistent judgement, different passing requirements. Students get inconsistent training/expectations/etc. as a result. Poor advising and not enough time to give students. |
| Challenges child code- Many Requirements | The program as a whole or generally has too many requirements and/or not enough support to meet those requirements - shouldn’t include why we got rid of something, just current issues. If one element is described as problematic, that should be issue with elements |
| Challenges child code- Master's Degree not valued | Master’s degree being seen as a “second best” option. |
| Challenges child code- Mental Health | Any mention of students being impacted by mental health. This includes being overwhelmed, something being traumatic, being stressed. |
| Challenges child code- Not preparing students for job market | The program is too specialized or missing big skills for the job market. A program setup not supporting or discouraging someone from pursuing a career. Also, not knowing the job market for the students is a way that we may not be preparing them. |
| Challenges child code-  Student intrinsic problems | People switch, want to do a master’s, or don’t want a PhD anymore, change goals, or personal life events |
|  |  |

**Table 3 Organization of codes into themes**

The below table is an overview of the how every individual code was organized into themes.

| **Theme** | **Description** | **Associated codes** |
| --- | --- | --- |
| **Theme One: Balance**  **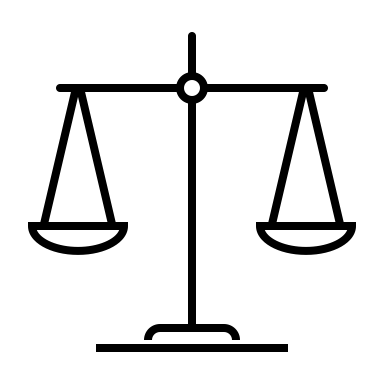** | Universities and faculty have a variety of responsibilities, causing dissonance between what is best for the education of the student and how to best meet other obligations. | - Challenges – issues with faculty and mentorship - Misalignment – What is best for students and what is best for other entities - Challenges - Issues with elements - Challenges – infrastructure - Challenges – masters’ degree not valued |
| **Theme Two: Assessment**  **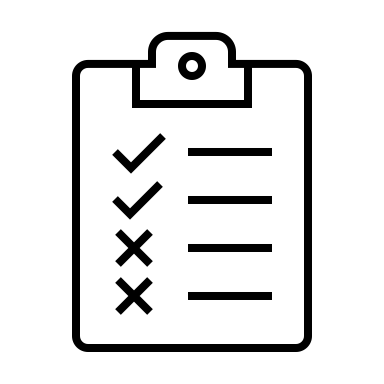** | Graduate programs lack a consistent way to assess success, meaning current practices are based primarily on anecdotal experiences and effectiveness is questionable. | - I don’t know – how to support my claims about DEC - How changes are made - Challenges – questionable preparation for job market - No way to assess success |
| **Theme Three: Implementation**  **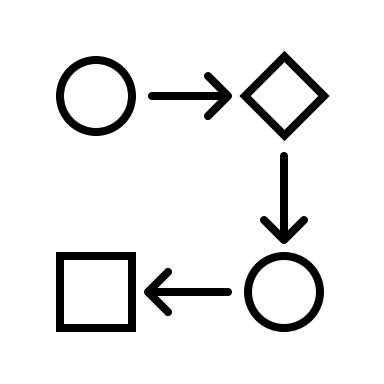** | There are a variety of issues with the implementation of programmatic elements of DEC that may undermine the goals of DEC and/or individual elements thereof. | - Challenges – issues with elements - I don’t know –what my coworkers are doing - Challenges – Negative impacts on students’ mental health - Challenges – infrastructure - Challenges – excessive requirements burden students - Challenges – issues with graduate student recruitment - Challenges – master’s degree undervalued - Variability in students’ experiences - Misalignment – Between what is best for students and other entities |
| **Theme Four: Mentorship**  **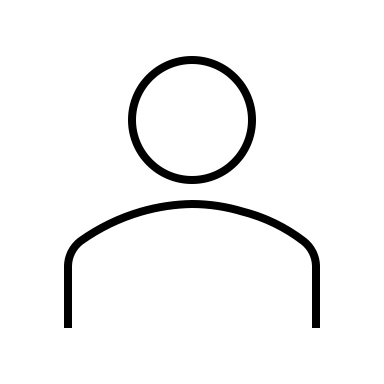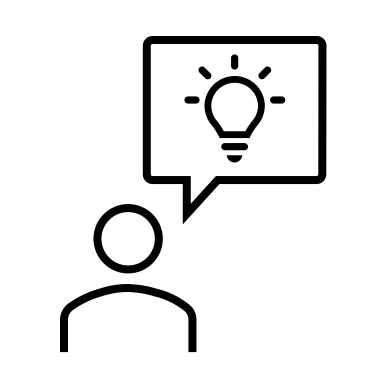** | Issues with faculty perspectives and practices relating to mentorship negatively impact the student. | - Challenges – issues with faculty and mentorship - Meta-awareness - Misalignment – Between faculty - Misalignment – Between what is best for students and other entities. |
